# Perceptions, expectations and challenges among men during in vitro fertilization treatment in a low resource setting: a qualitative study

**DOI:** 10.1186/s40738-019-0058-8

**Published:** 2019-07-04

**Authors:** Daniel Zaake, Anthony Kayiira, Imelda Namagembe

**Affiliations:** 1 Life Sure Fertility and Gynaecology Centre, Kampala, Uganda; 2 Uganda Martyr’s University Postgraduate Medical School, Kampala, Uganda; 3 St. Francis Hospital Nsambya, Kampala, Uganda; 4 Makerere University College of Health Sciences, Kampala, Uganda

**Keywords:** In vitro fertilisation, Fertility, Men’s perceptions, Low resource setting, Challenges

## Abstract

**Introduction:**

Partner support is very important in alleviating the burden of infertility related stress and although understudied, partner coping patterns also play a key role in the other partner’s ability to cope with the infertility experience which eventually affects treatment outcomes. Very few studies more so in a low to middle income setting, explore the psychological and social aspects of infertility in men. There is a need for a deeper understanding into men’s perceptions, expectations and challenges of fertility treatment in our low resource setting.

**Objective:**

To explore men’s perceptions, expectations, challenges and experiences during IVF treatment among men in a low resource setting.

**Methods:**

A qualitative research design was utilised. The study was conducted at life sure fertility and gynaecology centre. The study participants were men participating in the IVF cycles. They were selected purposefully by maximum variation sampling. All the interviews took place on the day of enrolment for treatment and inductive content analysis was used to draw meaning from the transcripts. Ethical approval for the study will be sought from Nsambya Hospital IRB/REC.

**Results:**

Seven major themes arose, and these included: (1) Societal influence on IVF treatment experience; (2) Social support during IVF treatment; (3) Feeling insignificant; (4) Financial burden; (5) IVF as an emotional bridge; (6) Inadequate sensitization; (7) Fear of treatment failure.

**Conclusion:**

Men’s experiences during IVF treatment were negatively affected by the society’s perceptions of IVF treatment and infertility, cost of treatment, perceived men’s involvement and insufficient knowledge about the IVF process. However, spouse and friends’ support helped with coping and the IVF treatment experience strengthened emotional bonds.

**Electronic supplementary material:**

The online version of this article (10.1186/s40738-019-0058-8) contains supplementary material, which is available to authorized users.

## Background

Infertility is defined as a failure to achieve a successful pregnancy after 12 months or more of appropriate, timed unprotected intercourse or therapeutic donor insemination [1]. Nonetheless, earlier evaluation and treatment may be justified after 6 months for women over age 35 years [1].

Male factor(s) are responsible for infertility in about 50% of couples that seek treatment for infertility [2]. That said, men can also be affected by infertility through being the partner of a woman who is infertile or being part of a couple with unexplained infertility [3]. Infertility is well known stressful process for both men and women [4–6]. Furthermore, those couples that seek fertility treatment particular In vitro fertilisation (IVF), experience emotional stress [7, 8] which is worsened by unsuccessful treatment [9]. Common reactions during IVF are anxiety and depression, while after an unsuccessful IVF, feelings of sadness, depression and anger prevail. After a successful IVF-treatment, IVF-parents experience more stress during pregnancy than ‘normal fertile’ parents [10].

Psychological stress may affect IVF cycles in one of two ways: first, there is some evidence that psychological distress might cause dysregulation in the uterus microenvironment and influence the ability to conceive [11]. A meta-analysis also found that interventions aimed at improving coping in IVF cycles were associated with better pregnancy outcomes [11]. The second way psychological stress affects IVF cycles is by influencing drop out and treatment termination rates [7, 12].

Evidence shows that partner support is very important in alleviating the burden of infertility related stress and thus male partners should be involved throughout the whole treatment process [4]. In addition, social and family support influences infertility treatment [12, 13], therefore health professionals should explore the quality of social networks and encourage seeking positive support from family and partners [13]. Lastly, although understudied, partner coping patterns also plays a key role in a the other partner’s ability to cope with the infertility experience [5].

Despite extensive research demonstrating that experiencing infertility is physically and psychologically stressful in all cultures and societies, most of these studies are focused on women [3, 14]. Very few studies more so in a low to middle income setting explore the psychological and social aspects of infertility in men [3]. It is well known that men and women experience infertility differently [3] and by extension this disparity would exist during infertility treatment. In spite overall limited data of the subject, one recent study on the male experience of infertility treatment done in a high income setting, found that men experience infertility as a mentally, physically and socially demanding condition [15]. A review by Jane RW Fisher and Karin Hammarberg (2012), found gaps in knowledge from available literature about factors governing seeking, persisting with and deciding to cease treatment; and infertility-related grief and shame among men. In addition, few resource limited settings had any data concerning men’s experiences of infertility and treatment [16]. In Uganda, assisted reproduction is a fast-growing field with no evidence pertaining men’s experiences of infertility and treatment in our setting. The studies available on men’s experiences are centred on pregnancy and childbirth [17].

There is a need for a deeper understanding into men’s experiences of infertility and fertility treatment especially in the face of a growing field of assisted reproduction in low income settings. This will enable us to develop contextualized and appropriate interventions towards improving men’s experiences during assisted reproduction. This will in turn positively impact their partner’s experiences, resulting in improved access and utilisation of assisted reproductive technologies in our low income setting. Therefore, this study aimed to explore men’s perceptions, expectations, challenges and experiences during IVF treatment.

## Methods

### Study design

A qualitative research approach is useful as it comprehends the dynamics of social life, the local people’s perspectives and their understanding [18]. The study commenced on 13th November 2016 and ended on 30th April 2017.

### Study setting

This study was conducted at Lifesure fertility and gynaecology centre. A private centre in peri urban Kampala, Uganda offering diagnostics and therapeutics in assisted reproduction on an outpatient basis. It runs 6 days in a week, 12 h a day. The centre is run by a team of two gynaecologists with speciality in reproductive medicine and a team of dedicated nurses for support. Many of the patients are referrals from hospitals and clinics. The number of couples attended to, are 10 per day. On average each batch of IVF cycles consists of 15 couples and 11 batches are run per year. At the initial and at every visit the couples are reviewed by the gynaecologist unless it’s a drug administration or drug refill.

### Study participants

The study participants were men aged 18 or more, participating in IVF cycles at the study facility and had given informed consent.

### Eligibility

Men were recruited for the study if they were part of a couple that had just completed an IVF treatment cycle for either a male factor related, female factor related or unexplained infertility problem. In addition, these men were recruited regardless of whether they had previous attempts at IVF and irrespective of previous IVF outcomes.

### Sampling

Participants were selected purposefully by maximum variation sampling to represent a variety of age groups, education level and socio-economic status. Sampling was done while analysing the interviews and continued until saturation was reached when 18 men had been interviewed.

### Data collection

Participants were approached by a nurse at the facility during IVF cycle enrolment and requested to participate in the study. All participants were assured of confidentiality, anonymity and that declining participation would not in any way alter the care they would receive. The nurses requested for permission to record the interview and the participants gave informed consent. The in-depth interview guide was reviewed by an expert in qualitative methodology and piloted on a set of eight men eligible for the study to assess acceptability, feasibility and content. The final version of the interview guide consisted of six open ended questions to allow a deeper insight into men’s perceptions, challenges and experiences. An example of the open-ended questions included “Can you tell about your experience during this IVF treatment process?” The questions addressed experiences during IVF treatment, social support, men involvement, interactions with fertility care provider and impact on relationship with partner. The complete interview guide is available as a Additional file 1. All the interviews took place after embryo transfer and two weeks before the pregnancy test. Privacy and confidentiality were maintained throughout the whole interview process. In-depth interviews were conducted by two trained IVF nurses with the aid of a voice recording device. One nurse asked the questions and the other documented all non-verbal communication. The in-depth interviews were conducted in English and Luganda with each interview taking an average duration of 45 min. The guiding questions were continuously reviewed of the by the researchers throughout the study process to ensure that the context was maintained. All participant identifiers were anonymized, and case report files secured under lock and key. Electronic data and audio recordings were kept in password protected files with data access restricted to the study authors.

### Data analysis

All the recorded data was transcribed, and the non-English transcripts translated into English. The qualitative data was analysed using inductive content analysis. Using an emergent strategy, to allow the method of analysis to follow the nature of the data itself, the transcripts were interpreted to identify patterns of words and phrases across the data that were important in describing their meaning. A group of words or phrases that related to the same central meaning were assigned codes inductively. Data coding continued until no new concepts emerged from successive coding. Similar or related codes were abstracted into themes. Verbatim quotations from interview transcripts have also used to illustrate relevant sub-themes. Handwritten notes were interpreted in the context in which they were written. WordStat® [version 8] software was used for data retrieval and triangulation of data during content analysis.

### Ethical considerations

Ethical approval was given by the St. Francis Hospital Nsambya REC; reference number 020.

All men participated voluntarily and gave written informed consent to be interviewed.

## Results

### Participant characteristics

Most of the men interviewed were between 31 to 50 years of age, in a monogamous marriage and having attained a tertiary level qualification with professional job. The average duration of infertility with their partners was 5.1 years with a few men having male-factor related infertility (27.78%) although majority had never fathered children. Slightly less than half of the men were having a repeat attempt at IVF. Participant characteristics are summarized in Table 1.

**Table 1:** Summary of participant characteristics

Characteristic	Frequency (%; *N* = 18)
Age category	
18–30	5.56
31–50	77.78
> 50	16.67
Marital status	
Married	100
Type of marriage	
Monogamous	83.33
Polygamous	16.67
Level of education	
Secondary	5.56
Tertiary	94.44
Occupation	
Unskilled worker	5.56
Skilled worker	11.11
Businessman	16.67
Professional	66.67
Duration of infertility ^a^ [years; mean SD]	5.1 ± 2.0
Fathered any children	
Yes	22.22
No	77.78
Male-factor related infertility	
Yes	27.78
No	72.22
Previous IVF attempt	
Yes	44.44
No	55.56

^a^: with current partner

### Emergent themes and subthemes

The perceptions, expectations and challenges among men in IVF treatment converged into the following themes and subthemes, as summarized in Table 2 below. A description of each theme is followed by a verbatim quote(s) to best depict the phenomenon. The participant identification number from which the quote was transcribed is included at the end of each quote.

**Table 2:** Summary of themes and subthemes obtained from content analysis

Theme	Subthemes
1. Societal influence on IVF treatment experience	Stigma associated with seeking IVF treatment Loss of masculine identity
2. Social support during IVF treatment	Spousal support Support from friends
3. Feeling insignificant	Biased encounters with IVF staff Undefined roles
4. Financial burden	
5. IVF as an emotional bridge	
6. Inadequate sensitization	
7. Fear of treatment failure.	

### Societal influence on IVF treatment experience

The societal perceptions about IVF treatment greatly influenced men’s experiences and were presented in two themes; Stigma associated with seeking IVF treatment and loss of masculine identity.

#### Stigma associated with seeking IVF treatment

Participants discussed the societal perception of seeking IVF treatment, as an acknowledgement of being infertile. This in addition to the society’s expectation, which came from family and friends, that married couples needed to have children elicited feelings of stigma. Participants opted to partake IVF treatment in secret, cautiously guarding their prescriptions and treatment information. Furthermore, they avoided encounters with family and friends during the IVF treatment process for fear of being stigmatised as depicted by a respondent below:

*“We have tried to have children naturally for 9 years…. Fearing the opinion of some friends, we opted for IVF in secret. We have not told anyone about our treatment, not even our parents…. When my wife had side-effects of the drugs we stayed home and did not attend any public functions because we feared being asked many questions. When people know the you are doing IVF, they label you infertile and secretly gossip about your misfortune*.” *(participant 1)*

#### Loss of masculine identity

A subset of participants with male-factor related infertility, reported that the role of a man was to make his wife pregnant. The fact they needed IVF treatment, made them feel they had failed their wives. This, compounded with the underlying stigma associated with IVF treatment, made men feel intensely inadequate and less masculine. This in turn negatively impacted their interactions with peers. The verbatim quotes below attempt to depict this subtheme:

*“When the doctor told me that I have low sperm count and IVF was the only way I could get my wife pregnant*…*I felt destroyed*… *less of a man. How would my friends see me? I almost left my wife because I did not see my value to her. But by the grace of God she was very understanding, and we agreed to start the treatment.” (participant 10)*

*“I have been through several relationships but failed to get any of the women pregnant. I had myself tested and I was told I had no single sperm and I would need a sperm donor to get my wife pregnant. I really don’t feel like I am a man anymore. I cannot tell my parents or any of my friends because I fear the embarrassment. When my friends talk about their children I keep quiet. I feel bad that I need another man to get my wife pregnant*…*But I love my wife and I want her to have children. That is why I decided to do IVF.” (participant 14)*

### Social support during IVF treatment

Men’s perception of support from their wives and friends influenced their IVF treatment experience and was captured in two subthemes: spousal support and support from friends.

#### Spousal support

Participants acknowledged that their wives were very understanding and offered them comfort throughout the long duration of infertility and the IVF treatment process. This was true particularly for those men whose wives had no other option but to turn to IVF and for men with male-factor related infertility. Some of the accounts are depicted below:

*“This is the second time we are attempting IVF. It has been a challenging experience, but I wanted my wife to have children because she doesn’t have any. She has always been there for me*…*Motivating me to keep trying.” (participant 2)*

*“I am the reason we can’t have children. But my wife has been understanding reminding me to take my medicine and not judging me. She has been with me at all the doctor’s appointments. We have been doing everything together throughout the treatment process. She even came to the room to help me during semen production.” (participant 6)*

#### Support from friends

There was wide agreement among participants that their friends who had gotten children through IVF frequently talked to them, shared their experiences and advised them. This made men feel they were not alone, and it encouraged them to persevere through the IVF treatment process, as depicted by the respondent below:

*“My close friends had challenges with having children and had gotten a baby through IVF. They approached me and advised I give it a try in order to end our search for a baby. They have kept in touch with me and my wife offering guidance and support. If it wasn’t for them I don’t think I would have gone through with IVF, it is so stressful.” (participant 12)*

### Feeling insignificant

The subthemes; Biased encounters with IVF staff and undefined roles, portray a perception of feeling insignificant, voiced by the participants in their experiences during IVF treatment.

#### Biased encounters with IVF staff

Participants perceived that the focus of the IVF treatment, was their female partners. From the encounter with the IVF staff to the treatment schedules all emphasis was made towards their partners. Participants also reported feeling out of place in the consultation room, as depicted by the accounts below:

*“During the initial consultation, most of the questions and investigations were directed to my wife. I was only asked if I had children and how often I had sex with my wife. In my mind I wondered*…. *why did I come along? When the treatment schedule was given to us, I noticed it had only my wife’s particulars*…. *I really felt unimportant. The doctor should talk more to husbands about the IVF program.” (participant 13)*

*“Whenever changes were made to the treatment schedule, my wife was called. I never received a single phone call. My wife knew more about the treatment process than me. I never received information directed toward me except when my wife was told to remind me to come and produce sperms…. The problem is that less information is given sometimes, and men are not involved actively.” (participant 2)*

#### Undefined roles

Overlapping with the subtheme of biased encounters with IVF staff, participants perceived the role of the man in the IVF treatment process was not properly defined. They felt that being responsible for paying the IVF fees and providing semen was insufficient. Participants needed to feel more engaged in the treatment process as depicted below:

*“After agreeing to start the program, the doctor told me I was needed only twice, once to carry out some blood tests and second to provide semen. I was not told how to support my wife during IVF except for paying the treatment fees. I still feel I did not do enough for her. Men need to be engaged more during the IVF process, so they do not feel left out.” (participant 4)*

### Financial burden

Overlapping with the subtheme of undefined roles, many participants perceived financial support as their role in IVF treatment. Men reported that the societal norms dictating men to be providers, put them under a lot of pressure to look for the necessary money. Men also reported that IVF treatment was very costly, and they had to give up their property in exchange for money to do the IVF. This caused them a lot of stress throughout the IVF process, as depicted by the respondents below:

*“From the onset of the IVF process, there has been a lot of emphasis on me to look for the money. I had to sell a plot of land that I had recently acquired to do IVF. When I failed to raise all the money at once, my wife called me constantly to finish our payment and this put me on a lot of tension. I hope it works out, otherwise I don’t think I can do this another time. IVF is too costly.” (participant 16)*

*“I love my wife very much but when she told about the cost of IVF treatment, I nearly said no. Then we were told to raise the money in a short period of time and that is when I felt like giving up on the whole process. I do not make a lot of money and certainly cannot afford IVF. So, I decided to take out a loan but throughout the treatment all I have been thinking about is how I am going pay that loan. I am worried.” (participant 18)*

### IVF as an emotional bridge

Participants perceived IVF treatment as an opportunity to bring them closer to their female partners. To most men, the process of supporting their partners through challenges of the IVF treatment strengthened their intimate bond as a couple as depicted by the respondent below:

*“We have been married for 8 years without children and I felt our relationship was beginning to fail. Doing IVF was our last chance to salvage our marriage. But going to the clinic together, looking for the money together and offering each other spiritual and moral support, has brought us closer.” (participant 11)*

### Inadequate sensitization about IVF

Participants discussed the lack of public knowledge about infertility treatment especially IVF treatment. Despite agreeing to go through the IVF treatment, participants still felt they did not completely understand the process of egg growth, semen production, embryo development outside the body and the low success rates. Other participants reported not knowing much of or being ready for the complications of the drugs used in IVF treatment. Participants also acknowledged that lack of sensitisation about IVF, stirred up several myths about the treatment such as the impact of IVF treatment on their partner’s risk for cancer and the risk for congenital abnormalities. Accounts of this theme are depicted below:

*“We were referred here for IVF treatment, but we did not know anything about the process. The doctor tried to explain to me, but everything was so new. I personally did not believe it was possible to grow babies outside the woman’s womb. I was so afraid to go through IVF. Please educate the public about IVF, to do away with stigma and fear about IVF. Also make it a point for women to come with their husbands.” (participant 5)*

*“I had some knowledge about test tube babies and when the doctor told me it was our only chance, I feared complications for my wife and giving birth to an abnormal baby. I talked to a friend and he warned me these drugs used to grow eggs might give my wife cancer, but we desperately needed a child. There is not a lot of public information about IVF and many times it is misunderstood.” (participant 3)*

*“When we agreed to start the IVF treatment, I was not aware that masturbation was required to produce sperm. The whole process of masturbation was a bad experience. It made me very uncomfortable. We need more information about the IVF process when enrolling into the program.” (participant 13)*

### Fear of IVF treatment failure

Participants considered IVF treatment to be a risky investment. They thought the odds of success were not favourable and yet the cost was too high. This overlaps with theme on financial burden and intensified men’s fears of IVF treatment failure. Men, especially those who had done IVF before, were worried about the emotional toll another failed IVF would have on them and their partners. Accounts of this theme are depicted below:

*“My experience has been so tough throughout the IVF process, someone has to be so strong to deal with sourcing the funds and the fear of not getting anything at the end!* … *The IVF process is very difficult.” (participant 16)*

*“This is the third time we are doing IVF. It has been a challenging journey and the fear of another failure is unmeasurable. We have done everything we were told. But I sometimes worry, weather it will work out, after all we have put in?... if this fails, it will take us very long to get over it”. (participant 6)*

## Discussion

In low-income settings, men’s experiences and perceptions during fertility treatment are largely undefined. This study explored men’s perceptions, expectations and challenges during IVF treatment at a fertility centre in Uganda. Overall, men were stigmatised by infertility and seeking IVF treatment, expected more involvement and information during treatment, and were stressed by the high cost of treatment. However, support from their partners and friends helped them cope with IVF related stress.

Societal perceptions on IVF treatment such as the interpretation of seeking IVF and masculinity greatly influenced men’s experiences during IVF treatment. In Uganda, much of the data relating to the psychosocial morbidity associated with infertility has been reported in women [19–21]. The limited data among men [22], pertains to the infertility state rather than seeking treatment as a focus of much of the social stigma. This study shows that in Uganda, seeking IVF treatment carries a similar risk of social stigma among men as would infertility. A possible explanation is that the pre-existing societal pressures and stigma facing men in a childless couple, were perceived to be validated by their decision to seek IVF. To these men accepting to do IVF meant they had totally failed their wives and the society, which considers IVF treatment as a confirmation of one’s infertility. As a result, men opted to do IVF in secret without the knowledge of close family and friends for fear being stigmatised. This perceived stigma limits social interaction and hinders social support which, according to Malina and Pooley [23], may negatively affect IVF outcomes.

Furthermore, Men with male factor related infertility in this study reported significant social stigma. This was largely caused their failure to reconcile the pre-existing societal expectation on men to impregnate their wives and their need for IVF to get children with their partners. As is common with other patriarchal societies [24], men in this study felt their masculinity was judged by their ability to get their wives pregnant which left them feeling less of men. The loss of masculinity and stigma associated with a male-factor related infertility has been reported by others [14, 15, 24].

This study found that wives were pivotal in driving the motivation to seek care and in helping men cope with the diagnosis and the IVF treatment process. This role of spousal support on coping with IVF treatment overlaps with the sub-theme on spousal support during IVF treatment and has been reported by Ying, Wu [25]. In their study partners offered emotional and physical support to each other which helped in coping with the stress of IVF treatment. In addition, friends who had undergone IVF were critical in helping men cope with the IVF treatment stress. This stresses the value of spousal support in coping with the emotional stress resulting from infertility and IVF treatment.

Interactions with the IVF treatment staff left men feeling insignificant during the treatment process as they felt excluded from care and thought care was biased to their partners with their roles largely undefined. This tendency of medical professionals to focus communication, fertility evaluation and care solely with the woman, has been reported by others [15, 26, 27]. This gender inequality in the infertility treatment experience causes discomfort to men whom many times are paying for the IVF treatment and strengthens the need for medical professionals to consciously support male involvement in IVF treatment.

Overlapping with the perception that men’s roles in IVF treatment were undefined, men saw IVF as a financial burden. This can be explained by the fact that Uganda, being a low-income country, has a GDP per capita averaging $600 [28]. Although 90% of the work force has some form of employment, men have a higher employment to population ratio and are still considered the primary providers in most households [29]. Unfortunately, a median monthly income equivalent of $58.75 with only 40% available after household consumption [29], cannot meet the $4000 average absolute cost of a single conventional IVF cycle. As narrated, many men resorted to selling their assets or mortgaging them against loans to meet the cost of IVF. This in addition to the perceived enormous pressure from societal expectations of men as providers, caused men significant distress. In addition, lack of men’s involvement in many sexual reproductive health activities, as reported by several African studies [17, 30, 31] and evidenced from the interactions with medical professionals, could partly explain why most men felt relegated to meeting financial costs of IVF treatment.

In Uganda except for the recent “Merck more than a mother” drive by Merk Serono® [32], there is still limited knowledge in the public especially among men about infertility and IVF treatment. A fact that was brought out clearly in this study. Men sighted limited information about aspects of IVF treatment process as a barrier to access. Lack of adequate information led to myths about IVF treatment risks and made men less prepared for the IVF process and outcome. As a result, some men recommended more public sensitization about the IVF process to reduce stigma and fear.

Extending from the theme on financial burden, was the fear of treatment failure which arose from men’s perception of IVF treatment as a risky investment, coming at a high cost with a low chance for success. This might have partly resulted from inadequate treatment counselling as was shown in a study by Jafarzadeh-Kenarsari [33]. Men in this study also expressed great worry about the consequent emotional impact of a negative result on them and their partners. This supports observations by other studies [9, 10, 34] that have highlighted the significant impact of negative infertility-treatment results on couples.

Nonetheless, this study found that men felt closer to their wives and their relationships were strengthened during treatment. This was similar to findings from other studies that outlined the potential for marital benefit during the couple’s infertility experience [27, 35]. This was true for couples that disclose their experiences and support each other [23].

This study brings to light how men in peri urban Uganda, a low income setting, view their IVF treatment experience. The predominantly negative perceptions such as social stigma, financial burden and unmet expectations during care serve as barriers to access. These negative experiences are areas where interventions should be focused to improve access and utilisation of fertility services. Some of the interventions to deal with these challenges may include but are not limited to; Targeted sensitisation programs these can be outreaches and mass media about infertility and IVF treatment. Training health workers in inclusive fertility treatment counselling. IVF cost subsidies through collaborations with the government and development partners.

The strength of this study lies mainly in the lack of data on the experience and perception of men during IVF treatment in a low income setting. This study was limited by the fact there was heterogeneity whether these men had previous IVF treatment and in previous IVF outcomes. There was also variation in presence of male-factor related infertility and whether one had fathered children, that could have biased some of the responses. This study was also limited by the inability to explore the effect of cultural diversity among participants on their perceptions and expectation of IVF treatment experience. Lastly, this study did not explore suggestions from the participants on how their participation and ultimate experience in IVF treatment can be improved.

## Conclusion

Men’s experiences during IVF treatment were negatively affected by the society’s perceptions of IVF treatment and infertility, cost of treatment, unmet expectations of their involvement and insufficient knowledge about the IVF process. However, spouse and friends’ support helped with coping and the IVF treatment experience strengthened emotional bonds.

## Additional file

Additional file 1: INTERVIEW GUIDE (Version 2.0). (PDF 146 kb)

## Data Availability

All data generated or analysed during this study is available from the authors upon reasonable request.

## Abbreviation

IVF: In vitro fertilization
