# Supplementary material for: Perceptions, expectations and challenges among men during in vitro fertilization treatment in a low resource setting: a qualitative study
Source: Fertil Res Pract. 2019 Jul 4;5:6. doi: 10.1186/s40738-019-0058-8 (PMC6609388; doi:10.1186/s40738-019-0058-8)
Supplement: Supplementary file 1 — INTERVIEW GUIDE (Version 2.0). (PDF 146 kb) [file 40738_2019_58_MOESM1_ESM.pdf]

Perceptions, expectations and challenges among men during In vitro Fertilization treatment in a low resource setting: a qualitative study

INTERVIEW GUIDE

(Version 2.0)

Date: |____|____|____|

Time: From ________ to __________

Note taker's code: ________________

Interviewer code: __________________

Respondent's code: _______________

1. Participant demographics

a) Age (years)
- 18-30 ☐
- 31-50 ☐
- > 50 ☐

b) Marital status
- *Single* ☐
- *Married* ☐
- *Cohabiting* ☐

c) Type of marriage
- *Monogamous* ☐
- *Polygamous* ☐

d) Educational level
- *None* ☐
- *Primary* ☐
- *Secondary* ☐
- *Tertiary/University* ☐

e) Occupation
- *Unemployed* ☐
- *Peasant farmer* ☐
- *Market Vendor* ☐
- *Secretary* ☐
- *Skilled worker* ☐
- *Self-employed/Businessman* ☐
- *Professional* ☐
- *Other* ☐

f) Duration of infertility in years (with current partner): ______________

g) Fathered any children
- *Yes* ☐
- *No* ☐

h) Male factor related infertility:
- *Yes* ☐
- *No* ☐

i) Number of previous attempts at IVF: _____

2. Guiding questions

a) Can you tell me about your experience along the path to IVF, as part of a couple with infertility?
b) Can you tell me about your experience during this IVF treatment process?
c) What kind of support did you receive during the IVF treatment process?
d) Can you tell me about your interactions with your fertility care providers?
e) What was your role during the whole IVF treatment process?
f) How has IVF treatment impacted the relationship with your partner?
